# Presenting symptoms predict local staging of anal cancer: a retrospective analysis of 86 patients

**DOI:** 10.1186/s12876-016-0461-0

**Published:** 2016-04-06

**Authors:** Matthias Sauter, Georg Keilholz, Helmut Kranzbühler, Norbert Lombriser, Meher Prakash, Stephan R. Vavricka, Benjamin Misselwitz

**Affiliations:** Department of Medicine and Specialities, Triemli Hospital, Zurich, Switzerland; Division of Gastroenterology and Hepatology, University Hospital Zurich, Rämistr 100, 8091 Zürich, Switzerland; Division of Radiation-Oncology, Triemli Hospital, Zürich, Switzerland; Department of Medicine and Specialities, Division of Gastroenterology, Triemli Hospital, Zurich, Switzerland

**Keywords:** Anal carcinoma, Symptoms, Physical examination, Tumor staging

## Abstract

**Background:**

Incidence of anal carcinoma (AC) is increasing and timely diagnosis is critical for efficient therapy. However, there is a paucity of recent studies addressing clinical symptoms and physical findings of anal carcinoma.

**Methods:**

We performed a retrospective study reviewing history, symptoms and physical findings from 86 patients with newly diagnosed AC. We analyzed frequency of symptoms and physical findings according to T and TNM stage and their predictive value regarding tumor stage.

**Results:**

Most patients presented with T2 (37 %) or T3 (29 %) cancer. 85 of 86 patients were symptomatic with anal bleeding (78 %), anal/perianal pain (63 %), weight loss (31 %) and foreign body sensation (22 %). 95 % of patients had ≥1 finding on physical examination including a visible tumor, palpable resistance and pain/blood during digital rectal examination. Patients with locally advanced disease (T3/T4) presented with more symptoms (*p* < 0.01) and more physical findings (*p* = 0.04) than patients with T1/T2 disease. On multivariate regression analysis perianal pain, painful defecation and weight loss were significantly associated with T3/T4 disease.

**Conclusion:**

Clinical symptoms and physical findings are present in nearly all AC patients. Pain referred to the perianal region, painful defecation and weight loss have predictive value for locally advanced disease.

**Electronic supplementary material:**

The online version of this article (doi:10.1186/s12876-016-0461-0) contains supplementary material, which is available to authorized users.

## Background

Anal carcinoma is an uncommon malignancy with an incidence of 2 new cases per 100,000 per year in the USA [1–6], comprising approximately 0.4 % of all tumors and 2.5 % of all gastrointestinal malignancies. Within the last decades the incidence of anal carcinoma has steadily increased and anal carcinoma incidence is now 2-fold higher than 30 years ago and 4-fold higher in selected subgroups of patients [3, 6, 7]. Risk factors associated with anal carcinoma are the lifetime number of sexual partners, receptive anal intercourse, cigarette smoking, genital warts and viral infections especially human papillomavirus (HPV), and human immunodeficiency virus (HIV) [8]. The increasing incidence of anal carcinoma might reflect changes in one or more of the risk factors mentioned above and might follow an increase in infection rates of HPV and HIV.

Timely diagnosis of anal carcinoma is critical, since for the treatment of early cancer highly effective and function preserving radio-(chemo) therapy is available. In contrast, the options for advanced and metastasized carcinoma are limited [1, 4, 9]. Clinical symptoms in the assessment anal cancer have only been insufficiently studied. Previous studies with a limited number of patients identified rectal bleeding (45 %), anorectal pain (20–35 %) and the sensation of a rectal mass (20–35 %) as the most prevalent complaints [10–14] with approximately 20 % of patients being free of symptoms in the largest study [10]. Clinical findings of anal carcinoma have not been summarized in detail. An association of symptoms or findings with early or an advanced disease has to the best of our knowledge not been tested. All studies were published between 1976 and 1986 and no recent data are available. In the intervening time medical practice has seen important changes including a massive increase in endoscopic evaluations of the colon. Following current guidelines, staging procedure of anal carcinoma now includes MRI and/or endosonography [1, 9], techniques unavailable 30 years ago. Considering these changes and the dramatic increase of anal carcinoma incidence, clinical presentation might differ now compared to the 1970s and the 1980s.

We therefore performed a systematic study of presenting symptoms and physical findings in patients with newly diagnosed anal cancer. Signs, symptoms and findings that differed for early and advanced anal carcinoma were identified.

## Methods

We performed a retrospective analysis of all patients referred to Triemli-Hospital, a tertiary care center and teaching hospital in Zürich, Switzerland from 1999 until 2013 for treatment of anal carcinoma. Patients were identified by an automated search within the internal clinical information system. Histological evidence of anal carcinoma was a requirement for inclusion and all patients with rectal carcinoma were excluded. We only considered symptoms and findings from the first presentation of anal carcinoma and recurrent anal carcinoma cases were excluded.

The study protocol was approved by the local ethics committee of Zurich county (Registration KEK-ZH 2010–0555). The study was performed according to the Declaration of Helsinki.

### Data collection

All relevant parameters regarding the initial clinical assessment including patient demographics, a detailed personal history, local and systemic symptoms, findings on physical examinations, and results of investigations (endoscopy with histology and staging investigations including CT, MRI and endosonography) were extracted from the documented patient history as well as from referral letters.

Tumor classification followed the 7^th^ edition of the American joint Committee on Cancer TNM staging [15] with T1 referring to a tumor size <2 cm, T2: a tumor between 2 and 5 cm, T3: a tumor >5 cm and T4 a tumor invading adjacent organs. N1 refers to lymph node metastasis in perirectal lymph nodes; N2 to metastasis in unilateral internal iliac and/or inguinal lymph node(s); and N3 to metastasis in perirectal and inguinal lymph nodes and/or bilateral internal iliac and/or inguinal lymph nodes. Stage I refers to a T1 tumor without lymph node involvement or distant metastasis. Stage II refers to a T2 or T3 tumor without lymph node or distant metastasis. Stage III refers to either i) T1-3 with N1 without metastasis or ii) any N2 or N3 positivity without metastasis independent of T-Stage or iii) T4 with N0 or N1 without distant metastasis. Stage IV refers to a tumor with advanced (N3) lymph node involvement (bilateral inguinal or bilateral internal iliac) or a tumor with distant metastasis. During our chart review all findings from all original investigations (CT, MRI and EUS) were evaluated and the appropriate tumor stage noted.

We also differentiated between distal, middle and proximal location according to i) digital rectal exam; ii) findings reported by CT, MRI or endosonography. Distal tumors included the anal rim/perianal region and proximal tumors included local invasion into the rectum.

### Data analysis

For each symptom and each physical finding we calculated the frequency for patients of each T-stage and TNM-stage. To identify differences for early and advanced carcinoma, *χ*^2^ significance of a trend (T1 – T4 or stage I – stage IV, respectively) was calculated using a generalized linear model.

To study the effect of the age at diagnosis on tumor characteristics our cohort was divided according to the median age (62 years) into a younger age group (≤62 years) and older age group (>62 years) and independent samples test was used for the comparison of means. A similar comparison was also performed to evaluate differences between the subgroups with proximal and distal anal carcinomas.

Multivariate logistic regression was used to analyze the predictability of early and advanced T-stage (or TNM stage) cancers. For this purpose T1-2 cancers (or TNM stage I and II) were categorized as early, T3-4 (or TNM stage III and IV) as advanced cancers. We tested clinical symptoms, physical findings and patient demographics such as gender, body mass index (BMI) and age at diagnosis as potential predictors. Potential predictors were sorted according to their *p*-values. In a step-wise procedure all descriptors with *p*-values <0.1 were eliminated and the regression analysis was repeated. This procedure eliminated several general descriptors including gender, BMI and age at diagnosis and the remaining variables with *p* < 0.1 and their corresponding odds ratios (OR) are reported. Similar analyses were also performed for the prediction of the TNM stage. All statistical analyses were performed using SPSS software.

## Results

Our electronic search identified 95 patients with anal carcinoma. 9 patients were excluded due to recurrent anal cancer and the analysis was restricted to the remaining 86 patients. All carcinoma cases had conclusive histology of anal carcinoma: 85 were squamous cell carcinoma, 10 of these with basaloid and one with cloacogenic subtype; one tumor was a neuroendocrine carcinoma with small cell features. No patient with anal adenocarcinoma was treated at our institution during the study period. No PCR data for HPV was performed.

Staging was done by MRI in 29 patients, CT scan in 71 patients and anorectal endosonography in 56 patients and most patients presented with T2 or T3 cancers (TNM stage II or III), respectively. Demographic data, T stage and TNM stage of the cohort are presented in Table 1. Anal cancer of the outer margin was significantly more frequent in men (*p* = 0.05). In contrast, cancer involving the anal channel tended to be more frequent in women (n.s.).

**Table 1:** Epidemiological characteristics of our patients with anal carcinoma

Description	Numbers
	Mean ± standard deviation
Gender	Male: 30 (35 %)
	Female: 56 (65 %)
Age	62 ± 13 years, range: 32–91 years
Diagnostic delay	8 ± 12 months, range: 0–62 months
BMI	24.7 ± 4.3 kg/m^2^, range: 17.3–34.7 kg/m^2^
Histology	Squamous carcinoma: 85 (99 %)
	Neuroendocrine carcinoma: 1 (1 %)
HIV	Positive: 5, Negative: 1
	Unknown: 80
T Stage	T1: 8 (9 %)
	T2: 32 (37 %)
	T3: 25 (29 %)
	T4: 21 (24 %)
TNM Stage	Stage I: 8 (9 %)
	Stage II: 27 (31 %)
	Stage III: 50 (58 %)
	Stage IV: 1 (1 %)
Tumor site involved	Distal anal channel: 43 (50 %)
	Middle anal channel: 37 (43 %)
	Proximal anal channel: 54 (63 %)
	Rectum: 36 (42 %)

### Clinical symptoms

Almost all patients reported symptoms due to anal carcinoma: 85 of 86 patients described at least one clinical complaint. The mean duration of symptoms before diagnosis was 8 months (median 3, range 0–62 months). The duration of the symptoms did not differ according to any of the symptoms, the total number of symptoms or tumor stage (not shown).

Clinical symptoms are summarized in Table 2. The most frequent symptoms were anal bleeding (78 %), anal/perianal pain (63 %, including 29 % with anal pain, 24 % with perianal pain and 38 % of patients with painful defecation), weight loss (31 %), tumor on self-palpation (26 %) and foreign body sensation (22 %). On average, patients described 3.3 symptoms.

**Table 2:** Clinical symptoms of patients with various tumor stages (expressed as percent of total number of patients with respective tumor stage). Statistical analysis: Generalized linear model

		All	T1	T2	T3	T4	*χ* ^2^
		*n* = 86	*n* = 8	*n* = 32	*n* = 25	*n* = 21	significance
Blood in stool		78 %	75 %	77 %	84 %	76 %	0.78
Pain	Painful defecation	38 %	25 %	26 %	52 %	48 %	0.26
	Anal pain	29 %	25 %	29 %	24 %	33 %	0.83
	Perianal pain	24 %	0	10 %	32 %	48 %	<0.01
Defecation and stool irregularities	Outlet obstruction	7 %	0	3 %	12 %	10 %	0.20
	Incontinence	12 %	0	6 %	16 %	19 %	0.07
	Pencil stool	6 %	0	6 %	4 %	10 %	0.45
	Diarrhea	11 %	0	10 %	16 %	10 %	0.50
	Irregular stool	7 %	0	6 %	8 %	10 %	0.40
	Constipation	6 %	0	0	8 %	14 %	0.02
Local mechanical symptoms	Foreign body sensation	22 %	0	2 %	20 %	29 %	0.28
	Pruritus	21 %	37 %	29 %	16 %	5 %	<0.01
	Tumor on self-palpation	26 %	25 %	26 %	24 %	29 %	0.81
Other organ involvement	Abdominal pain	5 %	0	0	4 %	14 %	0.02
	Mechanical ileus	1 %	0	0	0	5 %	0.16
	Vaginal stool	1 %	0	0	0	5 %	0.16
	Inguinal lymph nodes on self-palpation	2 %	0	3 %	0	5 %	0.64
Systemic symptoms/findings	Weight loss	31 %	25 %	20 %	30 %	60 %	<0.01
	Anemia	2 %	0	0	4 %	5 %	0.22
Asymptomatic		1 %	12 %	0	0	0	0.63
Total symptoms		3.3	2.12	2.75	3.52	4.43	<0.01

**Fig. 1:**
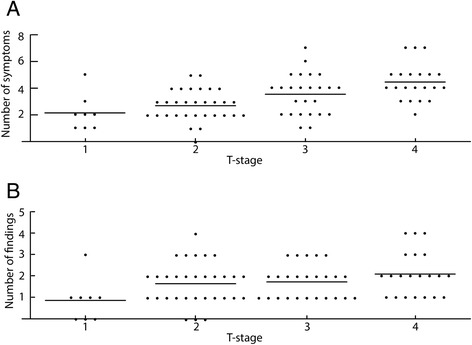
Relationship between the number of clinical symptoms (**a**) and physical findings (**b**) and the T-Stage of anal carcinoma. The line indicates average number of symptoms

Clinical presentation differed according to the T-stage of the tumor; patients with advanced disease described significantly more symptoms (2.1 for T1 and 4.4 for T2; *p* < 0.01, compare Table 2 and Fig. 1a). In addition, perianal pain, constipation, abdominal pain and weight loss were significantly more frequent in patients with locally advanced disease. Pruritus was more prevalent in patients with early T-stages. The most common symptoms anal bleeding and anal pain occurred with similar frequency in early and advanced tumors.

### Findings on physical examination

The vast majority of patients (82 out of 86) had pathological findings on physical examination (Table 3). Patients with locally advanced disease (T3 and T4) were likely to have more findings on physical examination than those with early disease (T1 and T2; Fig. 1b; *p* = 0.04). The frequency of all individual physical findings was similar in all tumor stages and no significant differences could be detected. Digital rectal examination was not possible due to pain in 2 patients, both of which presented with locally advanced carcinomas.

**Table 3:** Physical findings in our patients during digital rectal examination. Statistical analysis: Generalized linear model

	All	T1	T2	T3	T4	*χ* ^2^
	*n* = 86	*n* = 8	*n* = 32	*n* = 25	*n* = 21	significance
Painful palpation	39 %	25 %	30 %	40 %	65 %	0.15
Resistance on palpation	88 %	50 %	90 %	100 %	90 %	0.32
Blood on palpation	31 %	14 %	29 %	36 %	40 %	0.44
No clinical findings (or normal rectal examination)	7 %	37 %	9 %	0	0	0.02
Total number of physical findings	1.60	0.87	1.68	1.76	2.09	0.04

### Regression analysis

Multivariate logistic regression analysis was used to distinguish early carcinoma (T1 or T2) from locally advanced tumor (T3 or T4) using clinical symptoms and physical findings. We step-wise eliminated all variables which did not significantly contribute to T-stage prediction, using *p* = 0.1 as a cut-off. Our final model used 4 variables for T-stage prediction (Table 4). Thereby, *peri*anal pain was the single symptom with the strongest predictive value regarding the presence of locally advanced disease (odds ratio 6.1, 95 %-CI 1.5–25.1, *p* = 0.011). In addition, painful defecation and weight loss were able to predict locally advanced disease.

**Table 4:** Predictive value of symptoms for T-stage of anal carcinoma. The final model of our multivariate logistic regression analysis had significant predictive value to distinguish a localized vs. locally advanced tumor (*R* ^2^ = 0.37; *p* = 0.03). In the table the odds ratio (OR) of a symptom or physical finding for prediction of locally advanced cancer (T3/T4 vs. T1/T2) are shown. Only variables with *p* < 0.100 are indicated

Symptom or finding	OR	95 % CI	*p*-value
Perianal pain	6.1	1.5–25.1	0.011
Painful defecation	3.5	1.1–10.8	0.026
Weight loss	5.0	1.4–17.4	0.010
Pruritus	0.2	0.05–0.81	0.024

We also tested predictability of TNM-stage by symptoms and findings. According to our analysis TNM-stage could also be predicted, but with less precision (Additional file 1: Table S1). Perianal pain was the strongest predictor of TNM-stage, to a similar extent as described for T-stage.

### Age at diagnosis

To analyze effects of the age at diagnosis we divided our cohort into a younger (≤62 years, *n* = 42) and an older age group (>62 years, *n* = 44; Additional file 1: Table S2). Younger patients had a higher TNM-stage (2.7 vs. 2.3, *p* = 0.026) and a higher likelihood of proximal cancer than the older subgroup (chance of involvement of the proximal anal channel: 91 vs. 72 %, *p* = 0.044), but no further significant differences were detected and age at diagnosis is unlikely to confound our analysis.

### Location of anal carcinoma

Combining information available from imaging (MRI, CT-scan and endosonography) and the physical examination we determined for each patient whether the proximal anal channel (close to the rectum) and/or distal anal channel (bordering the skin) was involved by the carcinoma. Patients with proximal anal carcinoma had a higher T-stage than patients with distal cancer (mean T-stage 2.6 vs. 1.9, *p* = 0.03 for distal vs. proximal anal cancer, Additional file 1: Table S3). In addition, a description of distal carcinoma without proximal involvement was more frequent for localized cancers (OR for T-stage: 0.13, 95 % CI 0.03–0.64, *p* = 0.012) and proximal carcinoma was more common in locally advanced cancer (OR for T-stage: 3.0, 95 % CI 1.2–7.6, *p* = 0.019). However, multivariate logistic analysis did not result in any variables with significant contributions for prediction of early and advanced cancer.

## Discussion

In this study we provide a comprehensive clinical characterization of a cohort of patients with newly diagnosed anal carcinoma. According to our analyses, clinical symptoms have predictive value for local staging of anal carcinoma.

Our study fills a gap in our knowledge since no systematic study regarding physical findings in anal carcinoma has been performed. Furthermore, no contemporary study describing the clinical presentation of anal carcinoma is available and the incidence of anal carcinoma, the prevalence of risk factors and medical practice has tremendously changed since the publication of the last paper characterizing anal carcinoma almost 30 years ago [10–14]. In agreement with previous studies, bleeding, anal pain and sensation of an anal mass remain the most frequent symptoms of anal carcinoma. However, the presence of anal pain including painful defecation and perianal pain (63 vs. 20–35 %) as well as anal bleeding (77 vs. 45 %) were more frequent than in historical studies [10–14]. In addition we found that nearly all patients had at least one symptom likely associated with anal carcinoma. Truly asymptomatic anal carcinoma was exceedingly rare in our cohort (1 vs. 20 % in a previous study [10]). Pathological physical findings were also almost universal and only 6 % of our patients had a normal rectal examination.

Symptoms and findings of anal carcinoma were significantly correlated to the T-stage of the tumor. This predictive value of the clinical presentation has not been described previously and might be unique among gastrointestinal carcinomas (e.g. colon cancer, stomach cancer) for which early symptoms or findings are rare and symptoms do not reflect T stage or TNM stage [16–19]. For anal carcinoma, patients with advanced disease had a greater number of symptoms than those with early disease. They frequently reported perianal pain, changes in stool consistency and sometimes symptoms suggesting involvement of neighboring organs. Thereby, the predictive value of perianal pain for advanced tumor remained strong even in a multivariate regression analysis. This indicates that patients are able to “sense” local progression of this tumor. The ability of a significant fraction of patients with anal carcinoma to perceive locally advanced disease is likely related to the somatic sensation of the anal region, frequent mechanical stress during defecation and functional importance of the anal region for successful defecation. Similar to stomach and colon cancer, duration of symptoms does not predict tumor stage [16–18] but our study might be underpowered to detect subtle differences.

As a clinical message our data highlight the importance of a profound careful clinical history of patients with anal carcinoma. Attention should be paid to the localization of anal pain: According to our analysis, local anal pain or pain at defecation was compatible with both, early and advanced anal carcinoma. However, perianal pain (outside the anus) was almost exclusive found in patients with higher T-stages. Furthermore, the abundance of positive physical findings (in 99 % of our patients) underscores the importance of a careful examination of all patients.

Our study has limitations: i) data were collected retrospectively and 86 patients may be too small to detect all relevant associations. However, a prospective study of such a rare tumor would be difficult and the size of our study compares favorably to historic analyses and is the only study regarding this subject in almost 30 years. ii) No disease control group is available and all symptoms under discussion are not specific for anorectal cancer and might also be caused by hemorrhoids, fissure, infectious proctitis, rectal abscess, fistula or other common conditions [3, 20–23]. Therefore, our results cannot be used to estimate the diagnostic accuracy of individual symptoms for anal cancer. iii) Our analyses tested the predictive power of symptoms for T and TNM stage; however, we did not test the predictive power of symptoms and findings for overall survival or disease specific survival as hard clinical end points. Since in our follow-up analyses only 5 patients had died of anal cancer (not shown), our study is insufficiently powered for a survival analysis. iv) For the majority of our patients no result of an HIV test is available. Triemli Hospital is not a major referral center for HIV patients. We assume that for most of these patients HIV testing or at least a risk assessment has been performed with a negative result or a perceived low risk. Therefore, our results might not reflect the typical presentation of an HIV positive patient with anal carcinoma. v) No PCR testing for HPV was performed; with this method an HPV prevalence of 80–90 % has been described in other cohorts [24, 25]. vi) We did not perform a population-based study and therefore referral bias cannot be completely excluded. However, we estimate that the majority of patients with anal cancer of the 300,000–500,000 patients of the catchment area of our hospital were referred to the division of radiation oncology of this hospital for palliative or definite treatment. The number of 86 patients over a 10-year study period is in reasonable agreement with near-complete coverage if an incidence of 1–2 cases per 100,000 individuals is assumed [1–6].

## Conclusion

In summary, our study provides a contemporary summary of the clinical presentation of anal carcinoma. Nearly all patients displayed symptoms including pain, bleeding and foreign body sensation and/or physical findings such as pain on palpation, resistance and blood. Symptoms, especially pain perceived outside the anal channel, painful defecation and weight loss had predictive value for advanced disease. Due to the high sensitivity, functional importance and good accessibility of the anal and perianal region, clinical history and physical examination can identify patients with a high likelihood of advanced anal cancer.

## Additional file

Additional file 1: Table S1. Predictive value of symptoms for TNM-stage of anal carcinoma (similar to T- in Table 4). The final model of our multivariate logistic regression analysis had significant predictive value to distinguish early from advanced cancer (R2=0.40; *p*=0.040).The odds ratio of a symptom or physical finding for prediction of advanced cancer (stage III/ IV vs. stage I/ II). Only variables with p-values less than 0.10 are shown. **Table S2.** Characteristics of the older and younger subgroup of our anal carcinoma cohort. Statistical analysis: Mann-Whitney U test. **Table S3.** Characteristics of patients with involvement of the proximal (close to rectum) and distal (close to skin) anal channel. Statistical analysis: Mann Whitney U test. (PDF 233 kb)

## Abbreviations

AC: anal carcinoma
CI: confidence interval
CT: computer tomography
HIV: human immunodeficiency virus
HPV: human papillomavirus
MRI: magnetic resonance imaging
OR: odds ratio
