# Supplementary material for: Presenting symptoms predict local staging of anal cancer: a retrospective analysis of 86 patients
Source: BMC Gastroenterol. 2016 Apr 6;16:46. doi: 10.1186/s12876-016-0461-0 (PMC4822238; doi:10.1186/s12876-016-0461-0)
Supplement: Additional file 1: Table S1. — Predictive value of symptoms for TNM-stage of anal carcinoma (similar to T- in Table 4). The final model of our multivariate logistic regression analysis had significant predictive value to distinguish early from advanced cancer (R2=0.40; p=0.040).The odds ratio of a symptom or physical finding for prediction of advanced cancer (stage III/ IV vs. stage I/ II). Only variables with p-values less than 0.10 are shown. Table S2. Characteristics of the older and younger subgroup of our anal carcinoma cohort. Statistical analysis: Mann-Whitney U test. Table S3. Characteristics of patients with involvement of the proximal (close to rectum) and distal (close to skin) anal channel. Statistical analysis: Mann Whitney U test. (PDF 233 kb) [file 12876_2016_461_MOESM1_ESM.pdf]

**Variable**	**OR**	**95% CI**	p-Value
Perianal pain	13.3	2.2-69.0	0.002
Weight loss	4.9	1.4-16.8	0.011
Foreign body sensation	4.1	1.0-17.0	0.045
Irregular stool	11.5	1.0-124.0	0.043
Anal pain	3.0	0.90-10.1	0.046

Supplementary Table 1: Predictive value of symptoms for TNM-stage of anal carcinoma (similar to T- in Table 4). The final model of our multivariate logistic regression analysis had significant predictive value to distinguish early from advanced cancer (R^2=0.40; p=0.040).The odds ratio of a symptom or physical finding for prediction of advanced cancer (stage III/ IV vs. stage I/ II). Only variables with p-values less than 0.10 are shown.

	**≤62 years** n=42	**>62 years** n=44	**Significance** p-value
Average number of local symptoms	1.8	1.6	0.523
Average number of total symptoms	3.60	3.07	0.079
T-stage	2.8	2.5	0.137
TNM-stage	2.7	2.3	0.026
Involvement of proximal anal channel	0.91	0.72	0.044
Involvement of distal anal channel	0.64	0.63	0.928

Supplementary Table 2: Characteristics of the older and younger subgroup of our anal carcinoma cohort. Statistical analysis: Mann-Whitney U test

	Involvement of proximal anal channel<br>n=54	Involvement of distal anal channel<br>n=43	p-Value
Average number of findings	1.5	1.6	0.754
Average number of symptoms	2.9	3.3	0.485
T-stage	2.6	1.9	0.045
TNM-stage	2.4	1.8	0.022
Age at diagnosis (mean)	56	61	0.390

Supplementary Table 3: Characteristics of patients with involvement of the proximal (close to rectum) and distal (close to skin) anal channel. Statistical analysis: Mann Whitney U test
